# Changes in morphological traits, anatomical and molecular alterations caused by gamma-rays and zinc oxide nanoparticles in spinach (*Spinacia oleracea* L.) plant

**DOI:** 10.1007/s10534-023-00505-w

**Published:** 2023-05-12

**Authors:** Amina A. Aly, Gehan Safwat, Noha E. Eliwa, Ahmed H. M. Eltawil, M. H. Abd El-Aziz

**Affiliations:** 1 Natural Products Department, National Center for Radiation Research and Technology, Egyptian Atomic Energy Authority (EAEA), Cairo, Egypt; 2 Faculty of Biotechnology, October University for Modern Science and Arts (MSA), Giza, Egypt; 3 Genetic Department Faculty of Agriculture, Mansoura University, Mansoura, Egypt

**Keywords:** Gamma irradiation, ZnO-NPs, Morphological traits, Anatomy, SCoT, Molecular alterations, *Spinaciaoleracea*

## Abstract

Spinach seeds were irradiated with gamma-rays after that soaked in zinc oxide nanoparticles (ZnO–NPs) at 0.0, 50, 100 and 200 ppm for twenty-four hours at room temperature. Vegetative plant growth, photosynthetic pigments, and proline contents were investigated. Also, anatomical studies and the polymorphism by the SCoT technique were conducted. The present results revealed that the germination percentage was at the maximum values for the treatment of 100 ppm ZnO–NPs (92%), followed by 100 ppm ZnO–NPs + 60 Gy (90%). The application of ZnO–NPs resulted in an enhancement in the plant length. The maximum of chlorophylls and carotenoids content was recorded in the treatment, 100 ppm ZnO–NPs + 60 Gy. Meanwhile, the irradiation dose level (60 Gy) with all ZnO–NPs treatments increased proline content and reached its maximum increase to 1.069 mg/g FW for the treatment 60 Gy combined with 200 ppm ZnO–NPs. Also, the anatomical studies declared that there were variations between the treatments; un-irradiated and irradiated combined with ZnO–NPs plants which reveal that the leave epidermal tissue increased with 200 ppm ZnO–NPs in both the upper and lower epidermis. While irradiated plants with 60 Gy combined with 100 ppm ZnO–NPs gave more thickness of upper epidermis. As well as SCoT molecular marker technique effectively induced molecular alterations between the treatments. Where, SCoT primers targeted many new and missing amplicons that are expected to be associated with the lowly and highly expressed genes with 18.2 and 81.8%, respectively. Also, showed that the soaking in ZnO-NPs was helped for reducing molecular alteration rate, both spontaneous and induced by gamma irradiation. This nominates ZnO–NPs as potential nano-protective agents that can reduce irradiation-induced genetic damage.

## Introduction

Nanoparticles (NPs) technique appeared to have many advantages in agriculture at the start of the twenty-first century (Fraceto et al. 2016), and produced a surplus of 231 products in different agricultural fields (Rajput et al. 2021). This will increase the focus on this technology to avoid the use of chemical fertilizer for sustainable crop production and food safety to fulfil the food demand of the speedily rising worldwide population (Usman et al. 2020; Servin and White 2016). Nano-industry is largely depending on zinc-based NPs mainly in cosmetics industry (Carrouel et al. 2020), medicine (Anselmo and Mitragotri 2019), foods, and solar cells (Shende et al. 2021).

The affirmative and harmful effects of the use of ZnO–NPs on plant development are dependent on the used concentration (Rajput et al. 2021; Faizan et al. 2020). The achievement of nano-fertilizers is owed to the very small particles that are simply absorbed by plant roots. It is also, attributed to their large surface area that magnifies their benefits by increasing the plant sorption effectiveness (El-Saadony et al. 2021). Nanoparticle synthesis refers to creating small sized particles with favorable properties at dimensions lower than 100 nm, and thus may be carried out by chemicals or physical techniques. Nano-fertilizers increase the nutrient use effectiveness (NUE) by three folds and they also provide stress tolerance capability (Pruthvi Raj and Chandrashekara 2021).

The findings of the investigations that now exist show that various plant species react differently to nano-scale material. The yield development and yield attributes were owing to zinc, which is engaged in chlorophyll production throughout its impact on proteins, carbohydrates, and energy metabolism. Furthermore, if it is of nanosized the action speeds will be faster. Zinc ions are important for the activities of many enzymes and plants, as well zinc is required for chlorophyll biosynthesis which in turn contributes to theyield (Rahmani et al. 2016). Nanoparticles are atomic or molecular assemblies that have special physicochemical characteristics, like a high area to volume ratio, high antimicrobial activity, high photocatalytic reactivity, and a lower melting point, which are directly linked by their small size dimension of less than 100 nm (Zhang et al. 2019; Sabir et al. 2014). Plants are superior, and able to absorb chemicals substance in nanoparticle form than other, more conventional, and massive types because of their small size (Shende et al. 2021). The interaction between nanoparticles and plants cell, which cause both positive or negative morpho-physiological alterations, depend on the chemicals constitutes, sizes, shapes, the surface covering, reactivity, concentrations, and mode of nanoparticles application as well as the genotype, age, and developmental phase (Rajput et al. 2021; Torabian et al. 2016; Siddiqui et al. 2015).

Spinach (*Spinacia oleracea* L.) is a leafy vegetable crop that is commonly grown in open fields and kept environment throughout the Mediterranean basin. Due to its leaves' great dietary content and significant bioactive properties, it is a popular ingredient in many dishes and food products (Galla et al. 2016). Additionally, it has fewer calories but a higher concentration of bioactive compounds that exhibit strong antioxidant activity (Xu and Leskovar 2015). In addition to having a high concentration of minerals, spinach may also have a high concentration of anti-nutrients like nitrates and oxalates, which have serious negative effects on human health, and should be taken into account, along with appropriate agricultural practices which are usually applied to limit the levels of such contents (Hmelak Gorenjak and Cenciˇc 2013).

Gamma-rays are identified as affecting plant growth and causing cytological, genetically, physiologically, morphologically, and biochemical alterations in the plant tissues, these alterations depend on the dose intensity low or high (El-Beltagi et al. 2022; Aly et al. 2021a; Aly et al. 2019a, b).

Treating plants with NPs may lead to genetic variation depending on NP-size, type, and concentrations (Tymoszuk and Kulus 2020). Little is known about the genotoxicity of nanoparticles in plants, in order to achieve this, several of DNA-based methods, including Inter Simple Sequence Repeats (ISSR) and Random Amplification of Polymorphic DNA (RAPD), have been successfully used to accomplish this aim in different plants (Plaksenkova et al. 2019; Yang et al. 2018). The use of the SCoT marker would be significantly more effective than other random markers because of the longer primer distance and high annealing temperature. It is a polymorphism reproducible marker based on the short conserved regions in plant genes around the ATG translation start codon (Collard and Mackill 2009). The SCoT markers design did not need any specified genome sequence knowledge, making it possible to apply to plants that do not have genome references (Xiong et al. 2011). In the current study, SCoT polymorphisms have been created as the main marker to detect the diversity between the effects of gamma irradiation, ZnO–NPs at different concentrations, and their combinations.

Genetic relationships among a group of genotypes through molecular and phenotypic data representation may be performed utilizing multivariate techniques, which may intensify the information of many alleles and loci into several copied variables. Correspondence Analysis (CA) is one of the multivariate techniques that is an extension of the Principal Components analysis (Liu et al. 2022; Guo et al. 2022). This technique is described as a multivariate method to visualize categorical data in the graphical display using chi-square distances (Greenacre 1984). It serves as a supplementary analysis to the genetic distances matrix among different genotypes. Correspondence analysis tends to provide results like those obtained from cluster analysis dendrograms but is more informative and accurate than them, especially when there is a genetic exchange among close genotypes (Pissard et al. 2008). Also, CA is suitable for graphically exploring the relationship between two or more categorical variables and can lead to the stimulaing illustration of genotypes and loci as a cloud of points in metric spaces. Where it provides useful information about inertia or dispersion which is related togenetic diversity (Canon et al. 2001; Menexes and Koutsos 2021).

Therefore this investigation was carried out to evaluate the combined impact of gamma-rays at dose level (60 Gy) and ZnO–NPs at concentration (0.0, 50, 100, and 200 ppm) on morphological, anatomical as well as molecular diversity that occurred within the spinach plants using the SCoT marker.

## Materials and methods

Zinc oxide nanoparticles were purchased from Sigma-Aldrich as nano-powder with ≥ 99.5% metal basis.

### Irradiation treatments

*Spinacia oleracea* L healthy dry-seeds dash variety (spinach dash, Denmark) imported by Garra and Partners Company, Bab El-Khalk, Cairo-Egypt. The spinach seeds were packed in a polypropylene bag and then irradiated using gamma-rays (60 Gy) at a dose rate of 0.950 kGy h^−1^ at irradiation time. The irradiation treatments have been done at the Egyptian Atomic Energy Authority (EAEA), National Centre for Radiation Research and Technology (NCRRT), Cairo-Egypt utilizing the research irradiator (^60^Co Gamma cell 220).

The dose applied in this study was 60 Gy as well as the untreated seeds were served as control, prior to gamma irradiation the seeds were soaked in different concentrations of ZnO–NPs (0.0, 50, 100, as well as 200 ppm) for 24 h at room temperature (25 °C). The germinated seedling ratio of M0 seeds was evaluated at 15 days after sowing.

### Experiment design

This study was conducted as a field experiment in the greenhouse belonging to the Natural Products Dept., NCRRT, Cairo–Egypt, during the 2021–2022 winter season. Spinach seeds were planted in the greenhouse where they were spaced 50 cm apart and 30 cm between plants on both ends of the rows. All environmental requirements have been met, and agricultural needs have done. The study site's soil mechanical and chemical assessments are provided in Table 1.

**Table 1:** Physical and chemical properties of the experimental soil

A. Physical properties			
Sand%	Silt%	Clay%	Soil texture
57.3	21.2	21.5	Sandy clay loam

B. Chemical properties									
E.C. (ds/m)	pH	Cations (meq/l)				Anions (meq/l)			
		Ca^++^	Mg^++^	K^+^	Na^+^	CO_3_^=^	HCO_3_^−^	Cl^−^	SO_4_^=^
2.2	7.32	10.5	3.5	0.50	11.2	1.5	4.5	8.0	11.7

### Vegetative plant growth

The soaked seeds of (*Spinacia oleracea* L.) were grown utilizing conventional agricultural practices for two months, after that morphological characteristics were analyzed; plant length, leaf length, leaf numbers per plant, and roots length.

### Photosynthetic pigments

A spectrophotometric method was used to determine spinach leaf chlorophylls a, b, and carotenoids (Vernon and Seely 1966). Fresh leaf samples (0.5 g) were homogenized in a mortar with 85% acetone in the presence of washed dried sand and CaCo_3_ (Ca 0.1 g) in order to neutralize organic acids in the homogenate of the fresh leaves. The homogenate was then filtered through a sintered glass funnel. The residue was washed several times with acetone until the filtrate became colorless. The optical density of obtained extracts was determined using a spectrophotometer (Jasco model V-530, Tokyo, Japan) at 662, 644 nm for chlorophyll a and chlorophyll b, respectively, as well as 440.5 nm for carotenoids. The pigment contents were measured in mg/g FW**.**

### Proline content

Concentrations of proline were measured and the results were evaluated following the method of (Bates et al. 1973). In brief, 100 mg of frozen plant materials were homogenized in 1.5 ml of 3% sulphosalicylic acid and the residue was removed by centrifugation. Two ml glacial acetic acid and 2.0 ml acid ninhydrin reagent (1.25 g ninhydrin warmed in 30 ml glacial acetic acid and 20 ml 6 M phosphoric acid until dissolved) were added to 100 µl of the extract for one h at 100 °C and the reaction was then completed in an ice bath. One ml of toluene was added to the mixture, then warmed to room temperature and its optical density was measured at 520 nm. The amount of proline was calculated from proline standard curve in the range of 20–100 µg. The results were expressed as mg/g of proline equivalent for the fresh weight of the samples.

### Anatomical studies

Samples were fixed in a mixture of (10 ml formalin, 5 ml glacial acetic acid, 35 ml distilled H_2_O, and 50 ml ethanol 95%) no less than 48 h. After that, the samples were rinsed in 50% ethanol followed by dehydrating in normal butanol series. After that, they are embedded in paraffin wax of melting point of 56 °C. Leica RM2125 microtome has been used to section them to a thickness of 20 microns. They were then stained twice with safranin and fast green, cleaned in xylene, and at last mounted in Canada balsam (Nassar and El-Sahhar 1998). Photomicrographs were taken after a microscopic examination of the slides.

### DNA Extraction

Genomic DNA was isolated from the received fresh leaves (~ 100 mg) of each sample Using DNeasy Plant Mini Kit (QIAGEN, Santa Clarita, CA), genomic DNA was extracted from the obtained fresh leaves (about 100 mg) of each sample in accordance with the manufacturer’s procedure. The concentrations of isolated DNA were measured by a Nanodrop 8000 (Thermo Fisher Scientific Inc.).

### SCoT PCR and Data Analysis

The SCoT PCR amplification analysis was performed following Bhawna et al. (2017). A set of SCoT primers (Table 2) were screened against eight samples corresponding to each DNA sample and analyzed for molecular diversity. The PCR was carried out in a total reaction mixture of 25 μl comprising 1X PCR buffer, 2.5 mM MgCl_2_, 0.2 μM of every dNTPs, 2.0 μM of primers, one unit TaKaRa Taq™ DNA polymerase (Takara Bio Inc.), and 50–100 ng of genomic DNA. The PCR programme was set at 94 °C for 5 min for preliminary denaturation, followed by 35 cycles (94 °C for 1 min, 50 °C for one minute, and 72 °C for 90 s), and a final elongation at 72 °C for seven minutes. The amplification PCR outcomes were electrophoresed on a 1.5 percent agarose gel comprising ethidium bromide in 1X TAE buffer. A 100 bp DNA ladder-plus was developed. The PCR samples were organized in ascending mode (sample-one to sample-eight) through the loading on the agarose gel. The PCR results were visualized in UV light and gel image analysis and phylogenetics were gained with the Python-based tool called PyElph.

**Table 2:** SCoT primers utilized in the PCR and their nucleotide sequences

Primer name	Sequence
SCoT -13	ACGACATGGCGACCATCG
SCoT -14	ACGACATGGCGACCACGC
SCoT -24	CACCATGGCTACCACCAT
SCoT -33	CCATGGCTACCACCGCAG
SCoT -34	ACCATGGCTACCACCGCA
SCoT -61	CAACAATGGCTACCACCG
SCoT -66	ACCATGGCTACCAGCGAG
SCoT -70	ACCATGGCTACCAGCGCG
SCoT -71	CCATGGCTACCACCGCCG
SCoT -77	CCATGGCTACCACTACCC

### Molecular analysis

GelAnalyser3 software was used to analyze the DNA banding patterns generated by each primer. Obvious amplicons were scored as present (1) or absent (0) in a binary matrix for each primer. From this matrix, resolving power (Rp) was calculated, according to Prevost and Wilkinson (1999), and Polymorphic Information Content (PIC) was determined as described by Gorji (2011) method. Also, DNA-profile was done in accordance with the method of (Adhikari et al. 2015) with color discrimination for eight types of amplicons that illustrated molecular alterations caused by gamma-rays combined with ZnO–NPs from spinach plants. As well, correspondence analysis (CA) was done using XLSTAT 2019.2.2 (Addinsoft) to visualize the relationships among treated and untreated plants which summarizes the genetic distance among each combination depending on molecular and phenotypic data (Lam 2014; Canon et al. 2001).

### Statistic analysis

Results were stated as mean ± standard deviation and were analyzed by one–way analysis of variance (ANOVA). The mean values were compared at (*P* ≤ 0.05) by Duncan’s multiple (Duncan1955) to evaluate the significance between the treatments. Three replicates were utilized**.**

## Results

### Morphological traits

Nanoparticles (NPs) are organic or inorganic materials having sizes ranging from 1.0 to 100 nm; in recent years NPs have come into extensive use worldwide. Zinc is essential for crop nutrition as it is needed for a number of enzymatic reactions, metabolic functions, and oxidation–reduction reactions (Al Jabri et al. 2022).

Seeds of spinach plants tested by ZnO–NPs 50 and 100 ppm appeared significantly more likely to germinate with 90 and 92% respectively against 85% for control seeds. As well as the germination rate obtained from the irradiation dose 60 Gy was 90% as illustrated in Table 3.

**Table 3:** Morphological traits (germination percentage, plant length, leaves length, leaves number/plant and roots length) for spinach plants treated with ZnO-NPs at different concentrations and gamma irradiation or their combinations

Treatments		Germinated seeds (%)	Plant length (cm)	Leaf length (cm)	Leaves No	Root length (cm)
ZnO-NPs (ppm)	0	85 ± 2.65 ^b^	30.65 ± 0.38 ^d^	16.75 ± 0.49 ^d^	12.67 ± 0.58 ^ef^	7.40 ± 0.56 ^f^
	50	90 ± 2.00 ^a^	34.65 ± 0.43 ^c^	18.95 ± 0.38 ^c^	15.00 ± 1.00^ cd^	9.40 ± 0.62 ^e^
	100	92 ± 1.73 ^a^	43.40 ± 0.62 ^a^	20.12 ± 0.56 ^b^	20.33 ± 1.53 ^b^	10.65 ± 0.44 ^ab^
	200	85 ± 1.00 ^b^	41.24 ± 1.47 ^ab^	21.65 ± 0.44 ^a^	23.00 ± 0.00 ^a^	10.29 ± 0.50 ^bcd^
60 Gy + ZnO-NPs (ppm)	0	90 ± 1.00 ^a^	38.67 ± 1.81 ^b^	19.10 ± 0.56 ^c^	19.33 ± 0.58 ^b^	10.37 ± 0.47 ^abc^
	50	80 ± 0.00 ^c^	40.10 ± 2.79 ^b^	19.53 ± 0.82 ^bc^	16.33 ± 0.58 ^c^	11.17 ± 0.30 ^a^
	100	75 ± 1.73 ^d^	30.37 ± 2.35 ^d^	14.37 ± 0.51 ^e^	14.00 ± 0.00 ^de^	9.77 ± 0.25 ^cde^
	200	70 ± 1.00 ^e^	27.14 ± 2.21 ^e^	12.44 ± 0.35 ^f^	11.67 ± 0.57 ^f^	9.50 ± 0.11 ^de^

Results are expressed as means ± SD (n = 3), and means by various letter inside the same column are significantly difference (*p* ≤ 0.05)

The obtained results showed that the maximum plant length was observed for the treatment of 100 ppm ZnO–NPs (43.40 cm) followed by the treatment of 200 ppm ZnO–NPs (41.24 cm) as provided in (Table 3). The growth performances of the spinach plants in terms of leaf length showed that the greatest increase was found in the treatment 200 ppm ZnO–NPs (21.65 cm) followed by the treatment 60 Gy + 50 ppm ZnO–NPs (19.53 cm) as shown in Table 3. The best enhancement for the number of leaves per plant was observed for the treatment with 200 ppm ZnO–NPs (Table 3). Meanwhile, there was a slight increase in the root length for all the ZnO–NPs, 60 Gy, and their combinations treatments, and it was at the maximum increase for the treatment of 60 Gy + 50 ppm ZnO–NPs (Table 3).

### Photosynthetic pigments of spinach plants

Chlorophyll pigments have an important role in the photosynthesis process as they act as light-capturing centers. Changes in chlorophyll might adversely influence the plants growth. In addition, carotenoids, also essential plant pigments, have an important function in the protection of plant against biotic and abiotic stressors (Simkin et al. 2022).

**Fig. 1:**
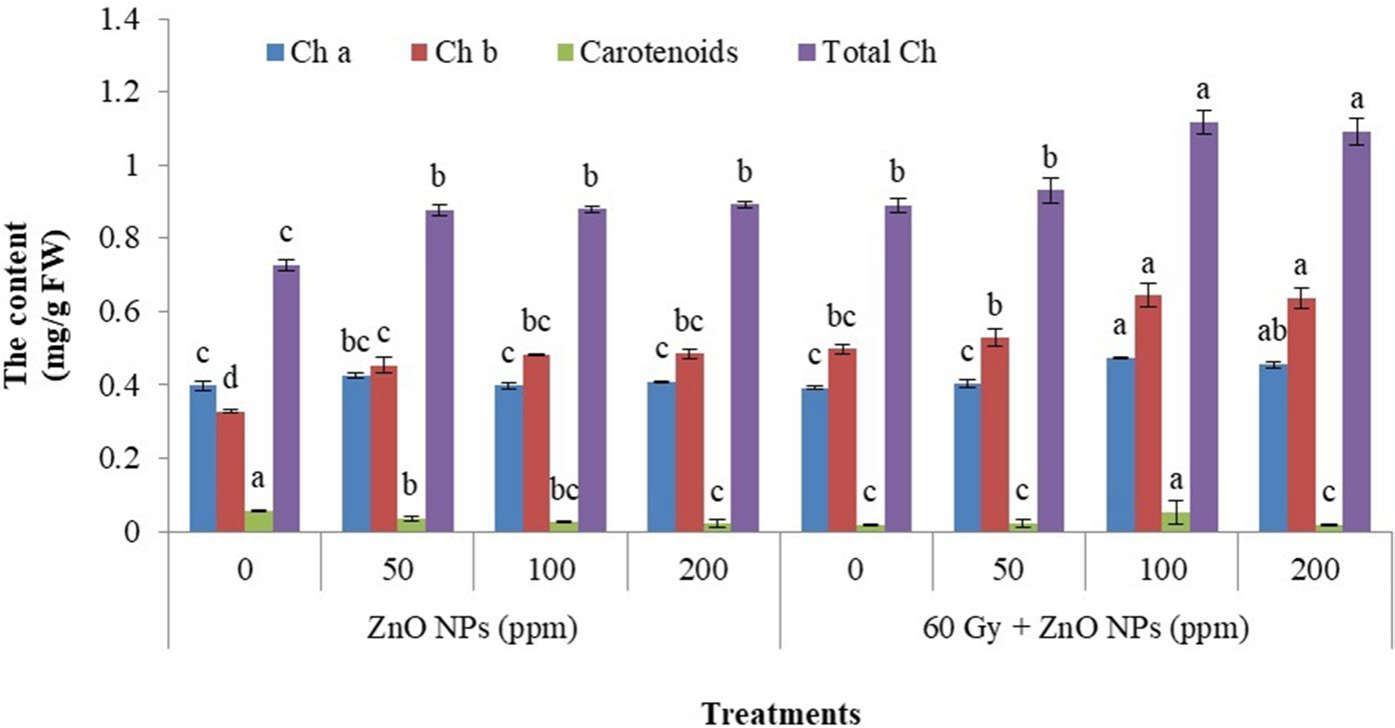
Chlorophylls **a** and **b**, total chlorophylls and carotenoids content for spinach plant treated with ZnO-NPs different concentration and gamma irradiation or their combinations. Vertical bars ± SD (n = 3) and varying letters on the bars in every sample are significantly differed at (*p* ≤ 0.05)

The results obtained for chlorophyll determination are shown in Fig. 1. As seen from these results, gamma irradiation combined with 100 ppm ZnO–NPs gave the greatest increase (0.474 mg/g FW) compared to control sample (0.398 mg/g FW) for chlorophyll a. The same trend was observed for chlorophyll b, the maximum increase was obtained for the treatment of 60 Gy + 100 ppm ZnO–NPs (0.644 mg/g FW) compared to the control (0.328 mg/g). Otherwise, the percentage of chlorophyll b increasing is higher than chlorophyll a. Meanwhile, carotenoids decreased due to increased concentrations of ZnO–NPs.

### Proline content

**Fig. 2:**
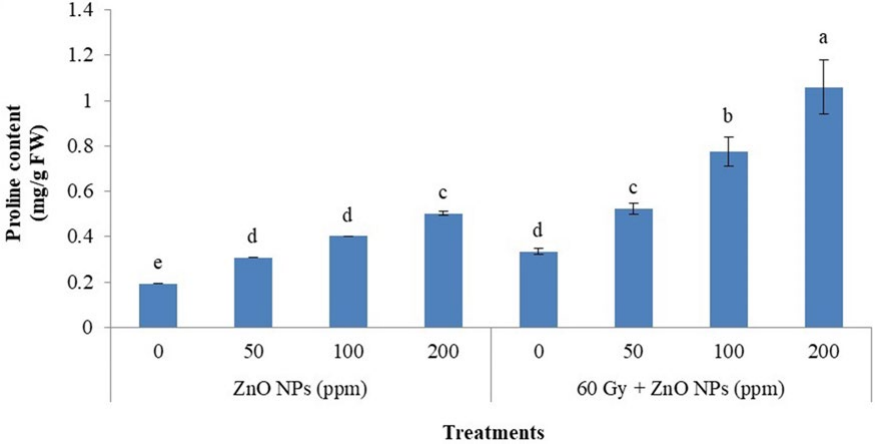
Proline content for spinach plants treated with ZnO-NPs at different concentrations and gamma irradiation or their combination. Vertical bars ± SD (n = 3) and varying letters on the bars in every sample are significantly differed at (*p* ≤ 0.05)

Proline is an essential organic solute and has an essential vital role in cell osmoregulation under stress. Proline content increased by increasing ZnO–NPs concentration, as well as when ZnO–NPs were combined with gamma irradiation (60 Gy), proline content was more than in ZnO–NPs individually and reached the maximum increase (1.059 mg/g FW) in treatment (60 Gy + 200 ppm ZnO–NPs) as outlined in Fig. 2.

### Leaf anatomical studies of spinach treated with ZnO-NPs at different concentrations and gamma irradiation or their combinations

**Fig. 3:**
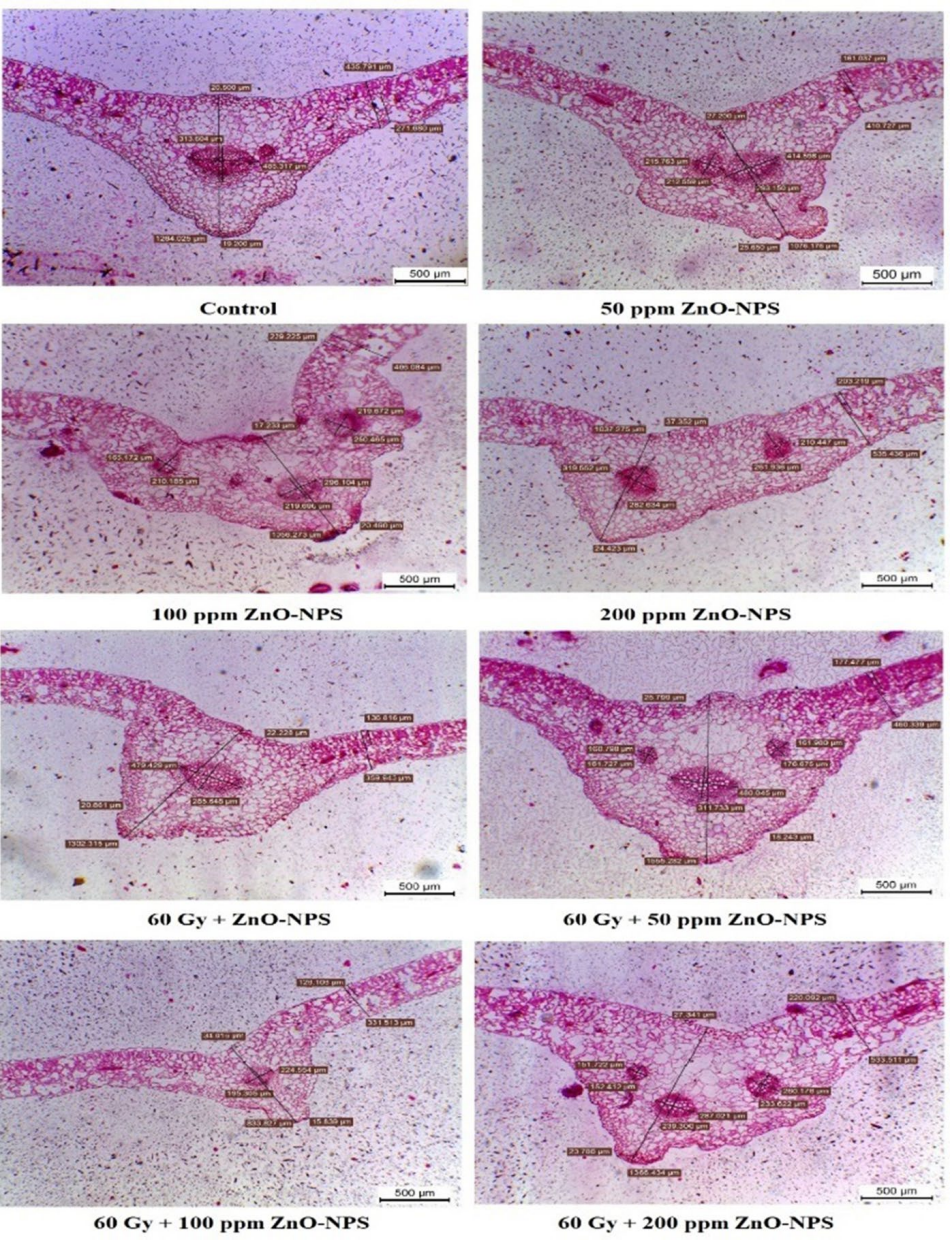
Leaf anatomy changings for spinach plant treated with ZnO–NPs different concentrations and gamma irradiation or their combinations

The anatomy of un-treated and treated leaves with gamma-rays (60 Gy), and ZnO–NPs at different concentrations or their combinations are provided in Fig. 3 and Table 3. The obtained results relived that epidermal tissue increased in both the upper and lower epidermis for the 60 Gy combined with ZnO–NPs 200 ppm treatment. While the sample 60 Gy combined with 100 ppm ZnO–NPs gave more thickness of the upper epidermis of spinach leaves. Regarding the mesophyll, the plaside tissue and sponge tissue showed the highest thickness with 100 and 200 ppm ZnO–NPs in un-irradiated and irradiated plants, respectively. A significant difference in the length and width of the bundles was also noted with increasing the concentration of ZnO–NPs in non-irradiated and irradiated plants (Table 4).

**Table 4:** Leaf anatomy (epidermis, mesophyll tissue and vascular diameter) for spinach plants treated with ZnO-NPs at different concentrations and gamma irradiation or their combinations

Treatment		Epidermis		Mesophyll tissue		Stele (vascular diameter)	
		Upper	Lower	Palisade thickness	Spongy thickness	Length	Width
ZnO-NPs (ppm)	0	20.800	19.200	164.111	271.680	313.604	485.317
	50	27.200	25.650	161.037	249.69	293.150 215.763	414.598 212.559
	100	17.233	20.490	229.225	236.859	210.185 219.690 219.672	165.172 296.104 250.465
	200	37.352	24.423	203.219	332.217	282.634 261.936	319.552 210.447
60 Gy + ZnO-NPs (ppm)	0	22.228	20.861	135.816	224.127	285.648	479.429
	50	25.799	18.243	177.477	282.862	161.727 311.733 161.980	160.798 480.045 176.675
	100	34.016	15.839	126.106	205.407	224.554	195.305
	200	27.341	23.786	220.092	533.511	151.722 239.300 233.622	152.412 287.021 260.176

### Molecular alterations assessment using SCoT molecular marker technique

**Fig. 4:**
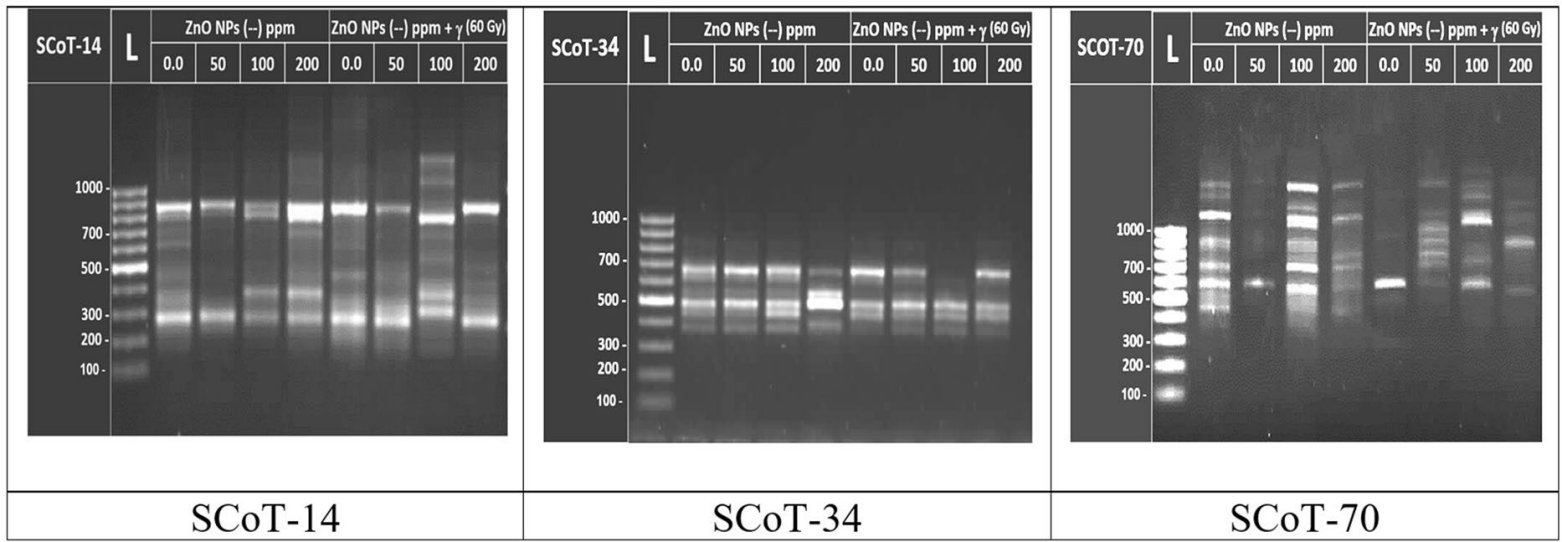
Banding patterns for SCoT-14, SCoT34 and SCoT-70 for examples of SCoT PCR -products. L, DNA ladder (100:1500 bp) and lanes from 2 to 9 represent the different treatments; lane 2: control, lane 3: 50 ppm ZnO-NPS, lane 4: 100 ppm ZnO–NPS, lane 5: 200 ZnO-NPS, lane 6: 60 Gy + 0.0 ppm ZnO-NPS, lane 7: 60 Gy + 50 ppm ZnO-NPS and lane 8:: 60 Gy + 100 ppm ZnO-NPS, lane 9: 60 Gy + 200 ppm ZnO-NPs

**Fig. 5:**
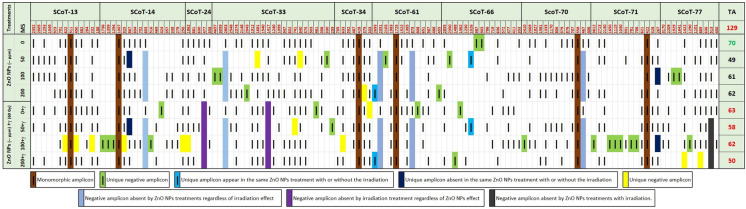
DNA-profile representation of SCoT Banding patterns based on 129 amplicons 6 of them were monomorphic (brown) and 55 of them were markers caused by the specific effects of soaking seeds in ZnO-NPs with or without γ irradiation in spinach plants. (in other various colors)

In this regard, SCoT markers were used to assess molecular alterations in spinach plants induced by soaking seeds in ZnO–NPs at 0.0, 50, 100 as well as 200 ppm concentrations with or without the irradiation at 60 Gy γ-rays. Initially, we should know that the primers used in this study (Table 2) target amplify SCoT-markers that are expected to be connected to functional genes. These primers often target highly expressed genes except for SCoT-13 and 14 which usually target lowly expressed genes (Sawant et al. 1999). In this study, through gel electrophoresis banding patterns and DNA profiles generated by SCoT molecular marker technique (Figs. 4, 5), this technique succeeded in revealing different patterns of DNA polymorphism among treated and untreated (control) of spinach plants. Where the DNA profile (Fig. 5) revealed and discriminate molecular alterations among these plants. This refers to the occurrence of alterations at the molecular level among spinach plants by soaking seeds in ZnO–NPs with or without γ-irradiation. Where ten SCoT-primers succeeded in amplifying a total of 129 amplicons of which 123 were reproducible polymorphic amplicons (94.9%) with a range between 188 to 4657 bp as shown in Table 5. These amplicons ranged from 5 for primer SCoT-24 to 23 for primer SCoT-33 with 100.0% polymorphism for these two primers. The SCoT primers were successful in targeting 55 amplicons that could be considered markers related to specific effects of applied treatments in comparison to the untreated (Fig. 5). On the other hand, the findings pointed out that the SCoT-33 primer showed the premier Rp value was 12.00 with polymorphism % and a PIC that was 100% and 0.353 percent, respectively (Table 5). Also, this primer was successful in targeting the highest number of SCoT-markers, a total of 11 markers, of which 6 are positive (appeared by specific effects of treatments) and 5 are negative (missing by specific effects of treatments) as provided in Table 5. This indicates the high informative and discrimination capability of SCoT-33 primer in the detection of reliable markers in spinach plants.

**Table 5:** Molecular results evaluated from banding patterns of SCoT molecular markers procedure

Primers	Molecular size range bp	TA	PA	Poly%	PIC	Rp
SCoT-13	188 : 2352	13	12	92.31	0.356	7.00
SCoT-14	250 : 1736	16	15	93.75	0.309	7.00
SCoT-24	358 : 1862	5	5	100.0	0.425	3.50
SCoT-33	259 : 4657	23	23	100.0	0.353	12.00
SCoT-34	333 : 705	7	6	85.71	0.254	2.25
SCoT-61	455 : 2693	13	12	92.31	0.341	6.50
SCoT-66	353 : 2855	15	15	100.0	0.363	8.00
SCoT-70	406 : 2410	13	12	92.31	0.385	8.00
SCoT-71	267 : 3612	13	12	92.31	0.291	5.25
SCoT-77	450 : 3170	11	11	100.0	0.335	5.50
All in one	188 : 4657	129	123			
	Average	12.9	12.3	94.9	12.3	6.50

TA: Total amplicons; Poly. %: Polymorphism percent.; PIC: Polymorphic Information Content; Rp: Resolving power; PA: Polymorphic Amplicon; A (-): Amplicons, appear in control and absent in one or more of the treatments; A ( +): Amplicons, absent in control and appear in one or more of the treatments; PA (-): Polymorphic A (-); UA (-): Unique negative amplicon, absent in one treatment; PA ( +): Polymorphic A ( +); UA ( +): Unique positive amplicon appear in one treatment

Findings in Fig. 5 and Table 6 showed molecular markers targeted by SCoT primers in this study that refer to genetic alterations caused by soaking seeds in ZnO-NPs different concentrations with or without irradiation. A total of 55 these markers, with different molecular sizes, 31 of them are positive (caused by a specific effect) and 24 are negative (missing by a specific effect). Some of these markers (UZ) indicate mutations induced by soaking seeds in one concentration of ZnO–NPs without γ-irradiation (a total of 12; 9 of them positive and 3 negative). While some other markers (Uγ) refer to mutations caused by γ-irradiation without soaking seeds in ZnO-NPs (a total of 5; 4 of them positive and one negative). Moreover, soaking seeds in one concentration of ZnO–NPs + γ-irradiation caused the largest number of markers (UZγ) a total of 24 markers (14 of them positive and 10 negatives). With higher specificity, soaking seeds in ZnO–NPs at 50 and 200 ppm with and without γ-irradiation caused appearing a specific marker for each treatment with molecular sizes 1026 and 2693 bp, respectivly. However, soaking seeds in ZnO–NPs at 50 and 100 ppm with and without γ-irradiation caused the loss of one amplicon (negative unique marker) for each treatments with molecular sizes of 267 and 867 bp, respictively. In addition, the soaking seeds treatments in ZnO–NPs, regardless of irradiation caused missing 5 amplicons, while γ-irradiation regardless treatments of soaking seeds in ZnO–NPs caused missing 2 amplicons. Whatever ZnO–NPs concentrations, the combination of soaking and irradiation caused missing one amplicon with molecular size of 513 bp to be targeted by the SCoT-77 primer.

**Table 6:** SCoT-markers caused by the specific effects of soaking seeds in ZnO-NPs with or without γ-irradiation in spinach plants

Primers	SCoT-markers (bp)													Total		
	UC		UZ		Uγ		UZγ		SAZ		NA					
	+	-	+	-	+	-	+	-	+	-	-Z	-γ	-Zγ	+	-	all
SCoT-13	-	-	-	-	-	-	-	3 (622, 450, 232)	-	-	-	-	-	-	3	3
SCoT-14	-	-	-	-	1 (424)	-	4 (1736, 1359, 1294, 516)	2 (925,250)	-	1 (867)	1 (603)	-	-	5	4	9
SCoT-24	-	-	-	-	-	-	-	1 (1862)	-	-	-	1 (477)	-	-	2	2
SCoT-33	-	-	4 (4657,3059, 2043, 330)	2 (1681,640)	1 (641)	-	1 (259)	1 (741)	-	-	1 (2803)	1 (1417)	-	6	5	11
SCoT-34	-	-	-	1 (379)	-	1 (333)	-	1 (633)	-	-	-	-	-	-	3	3
SCoT-61	-	-	2 (2103,455)	-	1 (808)	-	-	-	1 (2693)	-	2 (2351,875)	-	-	4	2	6
SCoT-66	2 (903,987)	-	1 (2350)	-	1 (1362)	-	1 (1488)	-	1 (1026)	-	-	-	-	6	-	6
SCoT-70	-	-	-	-	-	-	1 (2410)	-	-	-	1 (467)	-	-	1	1	2
SCoT-71	-	-	-	-	-	-	6 (3612,1650, 1115, 1030, 931, 781, 706)	-	-	1 (267)	-	-	-	6	1	7
SCoT-77	-	-	2 (1929,1641)	-	-	-	1 (1290)	2 (1422,889)	-	-	-	-	1 (513)	3	3	6
Total	2	0	9	3	4	1	14	10	2	2	5	2	1	31	24	55

UC( +): Unique amplicon appear in control.: UC(−): Unique amplicon disappears in control.; UZ( +): Unique amplicon caused by one ZnO-NPstreatment.: UZ(−): Unique amplicon missingby one ZnO-NPs treatment.; Uγ ( +): Unique amplicon caused by one γ-irradiation treatment.: Uγ (−): Unique amplicon missing byone γ-irradiation treatment.; UZγ ( +): Unique amplicon caused by one ZnO-NP s + γ irradiationtreatment: UZγ (−): Unique amplicon missing by one ZnO-NPs + γ irradiationtreatment.; SAZ ( +): Specific amplicon appear in the same ZnO-NPs treatment with or without the γ-irradiation.; SAZT (−): Specific amplicon missing by the same ZnO-NPs treatment with or without the γirradiation.; NA (-Z): Negative amplicon missing by ZnO-NPs treatments regardless of γ-irradiation effect. NA (-γ) Negative amplicon missing by γ-irradiation treatment regardless of ZnO-NPs effect; NA (-Zγ) Negative amplicon missing by ZnO-NPs treatments with γ-irradiation

**Fig. 6:**
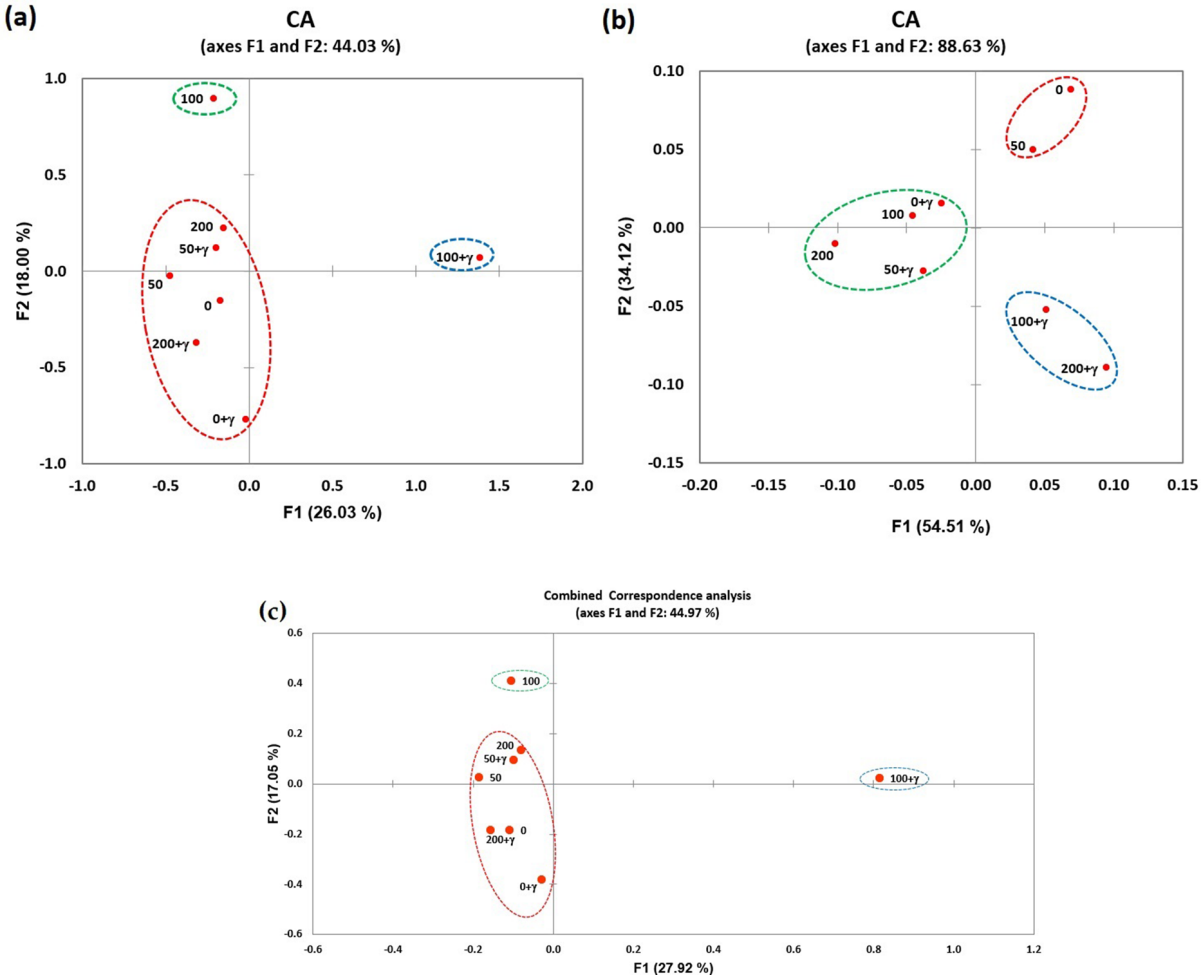
Correspondence analyses (CA) plot for treated and untreated spinach plants depending on binary data of SCoT molecular marker technique **a**, phenotypic traits **b** and their combination **c**, where the first two axes F1 and F2 contribute a total 48.48%, 88.6 3% and 44.97% of the inertia or dispersion for each analysis respectively

**Fig. 7:**
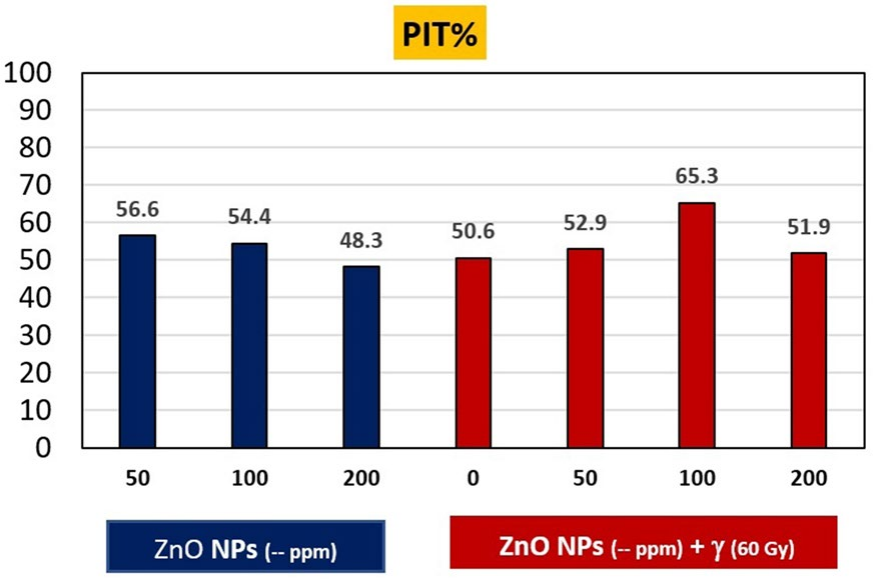
Polymorphism % induced by treatments (PIT %) detected by SCoT molecular marker technique in spinach plants

Commonly, to assess the variations caused by each treatment compared to the control, Correspondence Analyses (CA) was used to clarify the inertia or dispersion caused by the applied treatments (Fig. 6), as well as the polymorphism % for each treatment, compared with the control was computed (Table 7 and Fig. 7). Correspondence analyses were applied using the molecular and phenotypic data as well as their combination (Fig. 6a, b, and c, respectively). Where the molecular data were presented as a scatterplot (Fig. 6a) based on the first two axes (F1 and F2) which give the maximum input of inertia in the data matrix in a manner that can be regarded equivalent to the total variation in allelic occurrence (Benzecri 1992). The F1 as well as F2 axes, accounted for 34.93% and 13.55% of the variation, respectively; thus, 44% of the variance was accounted for the first 2 axes. Meanwhile, the treatments were partitioned into 3 groups distinctive by circles in agreement with the cluster analysis which was distributed in a range of molecular distances from 0.315 to 0.640 (data not shown). The first group CA1 (surrounded by red) included control and treatments except for 100 ppm ZnO–NPs and 100 ppm ZnO–NPs + γ-rays which were partitioned into two independent groups CA2 and CA3 (surrounded by blue and green respectively). The scatterplot obtained from the molecular data can compare with another scatterplot that was computed depending on morphological data (Fig. 6b) which was represented by two axes with 88.63% total contribution in variation. Where also harmonious with the cluster analysis which distributes in a range of phenotypic distances from 5.3 to 29.7 (data not shown). By comparing the two analyzes, it is clear that the treatments are distributed at the molecular scatterplot by a greater variation than their distribution at the phenotypic scatterplot. This led to the combined scatterplot (Fig. 6c) appearing with a distribution similar to that of the molecular scatterplot with total contribution rates of 44.97% for the two axes. Thus indicating that the treatments led to alterations at the molecular level that were different from alterations at the phenotypic level.

**Table 7:** Polymorphism for SCoT-amplicons among treatments and control

Treatment Primers	ZnO-NPs (–) ppm												ZnO-NPs (– ppm) + γ (60 Gy)																All	
	50				100				200				0 + γ				50 + γ				100 + γ				200 + γ					
	CA	NA	MA	PIT%	CA	NA	MA	PIT%	CA	NA	MA	PIT%	CA	NA	MA	PIT%	CA	NA	MA	PIT%	CA	NA	MA	PIT%	CA	NA	MA	PIT%	NA	MA
SCoT-13	6	3	3	50.0	6	3	3	50.0	6	1	3	40.0	8	2	1	27.3	9	0	0	0.0	2	1	7	80.0	9	0	0	0.0	10	17
SCoT-14	3	0	4	57.1	5	2	2	44.4	6	2	1	33.3	5	1	2	37.5	3	2	4	66.7	4	7	3	71.4	4	1	3	50.0	15	19
SCoT-24	3	0	2	40.0	3	0	2	40.0	3	0	2	40.0	2	0	3	60.0	3	0	2	40.0	1	0	4	80.0	2	0	3	60.0	0	18
SCoT-33	5	2	8	66.7	7	5	6	61.1	9	5	4	50.0	9	3	4	43.8	4	4	9	76.5	9	1	4	35.7	9	1	4	35.7	21	39
SCoT-34	5	0	1	16.7	6	0	0	0.0	4	0	2	33.3	3	1	3	57.1	5	0	1	16.7	4	0	2	33.3	4	1	2	42.9	2	11
SCoT-61	3	2	4	66.7	1	1	6	87.5	2	4	5	81.8	6	3	1	40.0	3	1	4	62.5	3	1	4	62.5	2	3	5	80.0	15	29
SCoT-66	1	2	4	85.7	1	3	4	87.5	1	1	4	83.3	1	5	4	90.0	1	4	4	88.9	1	4	4	88.9	2	2	3	71.4	21	27
SCoT-70	2	0	7	77.8	7	3	2	41.7	5	2	4	54.5	2	0	7	77.8	5	2	4	54.5	5	2	4	54.5	3	1	6	70.0	10	34
SCoT-71	4	3	0	42.9	1	0	3	75.0	4	1	0	20.0	3	2	1	50.0	4	2	0	33.3	1	8	3	91.7	4	2	0	33.3	18	7
SCoT-77	4	1	1	33.3	4	3	1	50.0	5	1	0	16.7	5	2	0	28.6	4	2	1	42.9	4	4	1	55.6	0	0	5	100.0	13	9
All in one	36	13	34	56.6	41	20	29	54.4	45	17	25	48.3	44	19	26	50.6	41	17	29	52.9	34	28	36	65.3	39	11	31	51.9		

*CA* common amplicon between treatment and control; *NA* new amplicon appearance by treatment; A: Missing amplicon by treatment; PIT %: Polymorphism% induced by treatment

Therefore, polymorphism percentage induced by treatments (PIT %) applied in this study was calculated to detect relative DNA variations in treated spinach plants compared to control. Depending on the appearance or disappearance (missing) of amplicons between each treatment and control. From Table 7 and Fig. 7 it is clear that the polymorphisms differed for each treatment from one primer to another and for each primer from one treatment to another. Where the soaking in 100 ppm ZnO–NPs + γ-rays was the highest in inducing PIT % (65.3%) through missing of 36 amplicons and the appearance of 28 new amplicons compared to control, 18 of them may be connected to lowly expressed genes and the rest amplicons may be associated with the highly expressed genes (De Vicente et al. 2004). Whereas the soaking in 200 ppm ZnO-NPs without irradiation was the lowest in PIT % (48.3%) through missing of 25 amplicons and the appearance of 17 new amplicon compared to control, 7 of them may be connected to lowly expressed genes and the rest amplicons may be associated with the highly expressed genes. On the other hand, the SCoT-33 primer targeted the largest total number of missing and new amplicons which were caused by applied treatments compared to the control (21 and 39 amplicons, respectively). While, the SCoT-33 primer targeted the fewest total number of missing and new amplicons (2 and 11 amplicons, respectively). These two primers target amplify SCoT-markers that are expected to be connected to highly expressed genes (Sawant et al. 1999). Whereas, the two primers which usually target lowly expressed genes (SCoT-13, 14) targeted a total number of MA and NA of 10, 17 for SCoT-13 and 15,19 for SCoT-14, respectively. Where the percentage of what was targeted by these two primers was 18.2% of the total MA and NA versus 81.8% targeted by another primers that are expected to be targeted to highly expressed genes.

In general, from Fig. 7, it is illustrated that the soaking treatments in ZnO–NPs led to a decrease in PIT % (indicates a decrease in molecular alteration rate caused by each treatment) with increasing ZnO–NPs concentration. Where the lowest value of PIT % was when soaking in 200 ppm (48.3%). In contrast, irradiation led to an increase in the PIT % with increasing ZnO–NPs concentrations upto 100 ppm (65.3%). whilst, the effect of soaking at 200 ppm was beneficial in decreasing the value of PIT % (to 51.9%), indicating that the soaking in ZnO–NPs was helpful in reducing molecular alteration rate, both spontaneous and induced by gamma irradiation.

Finally, it becomes clear that SCoT markers were effective in analyzing induced molecular alterations by treatments applied in this study.

On the other hand and overall, SCoT analysis results demonstrate that soaking seeds in ZnO–NPs with or without γ-irradiation altered the genome of treated spinach plants. Where many amplicons disappeared and new amplicons appeared in all ZnO–NPs concentrations compared to the control samples. Gamma irradiation increased from alteration rates until they reached the highest level at the concentration of ZnO–NPs 100 ppm. Nevertheless, increasing the concentrations of ZnO–NPs to 200 ppm with or without γ-irradiation had an effective action in reducing the molecular alteration rate and genomic template stability.

## Discussions

Due to the large surface area and small size, NPs are served as nano fertilizers with increased diffusions rate. They have greater dissolve capacities, which enable earlier nutrient availability to roots and, as a result, increases crops yield (Dey et al. 2018). Results of the recent study (Table 3) displayed that seeds priming by ZnO–NPs could enhance spinach development, under gamma irradiation stress. Previous studies confirmed useful evidence to support the beneficial effects of nano-sized zinc to plants growth such as corn (*Zea mays*) (Neto et al. 2020), cotton (*Gossypium hirsutum*) (Singh et al. 2021) and chickpea (*Cicer arietinum*) (Burman et al. 2013). Also, zinc is identified to be engaged in managing enzyme, proteins synthesis, cells elongation, modifiable membranes role, and improvement of plant resistance to environmental challenges (Cakmak 2008).

The low doses of gamma radiation showed improvement in roost and shoots length in mung bean (*Vigna radiate*) and pea (*Pisum sativum*) compared to the control (Atteh and Adeyeye 2022). In addition, gamma-rays showed high germination percentage and stimulate vegetative traits as plant height, leave numbers/plant, roots length and roots diameter (El-Beltagi et al. 2022). The same trend was found in red radish (*Raphanus sativus*-Red) blackberry (*Rubus fruticosus*), cowpea (*Vigna unguiculata*) and culantro (*Eryngium foetidum*) which were exposed to low doses of gamma-rays, respectively (Aly et al. 2023a, 2022; El-Beltagi et al. 2013; Aly 2010). The impacts of gamma-rays on germination might be attributed to the establishment of RNA or proteins synthesis (Abdel-Hady et al. 2008).

Nanoparticles applied to the broccoli led to seeds germination, roots length, shoots length, seedling weight leave numbers, plants height, and leaf area. Also, provided that ZnO-NPs when utilized to increase quantities did not produce any poisonous impact on plant and can be used as nano-fertilizer on a commercial basis (Awan et al. 2020). Zinc oxide nanoparticles significantly improved growth and photosynthetic effectiveness, and activate the antioxidant systems in tomato seedling (Faizan et al. 2018). Cotton (*Gossypium hirsutum*) plants tested by ZnO–NPs established a considerable enhancement in biomass, shoots and roots length as well as roots area and this improvement was raised by rising of the ZO-NPs concentration (Singh et al. 2021). There was a correlation between nutrients content, growth parameters and changing in nanoparticles concentrations. As it was obvious that some nanoparticles were more efficient with low concentrations while others gave positive effects at high concentrations. Furthermore, spray application of zinc oxide and titanium dioxide nanoparticles have a prospective effect for improving growth traits, photosynthetic effectiveness, and biochemical features of faba bean (*Vicia faba*) and broccoli (*Brassica oleracea*) plants respectively (Ragab et al. 2022; Aly et al. 2021b).

It was observed that ZnO–NPs treatments produced higher photosynthetic pigments in the current study (Fig. 1) and this is in accordance with the findings reported in the literature (Singh et al. 2021; Ragab et al. 2022; Ramegowda et al. 2013).

Previous study illustrated that treating lupine (*Lupinus*) seeds by ZNPs increased the chlorophylls and another photosynthetic pigments in plants, and that is attributed to the role of zinc as a crucial nutrient of plants. As zinc plays an essential role on plant metabolism by influencing the activities of vital enzymes like carbonic anhydrase. This enzyme is a metalloenzyme that increases the availability of carbondioxide for plants (Latef et al. 2017). At the concentration of 200 as well as 300 mg/l nano-zinc, decreased chlorophylls value by 50%; while, the carotenoids concentration did not change in the Arabidopsis plants (Wang et al. 2018). Moreover, the nano-zinc plays a key role in photosynthesis, affecting the action of enzymes like carbonic anhydrase and that is owing to its small size which affects its absorption so it influences chlorophyll concentrations and stomatal conductance (Ramegowda et al. 2013). On the other hand, ZnO–NPs decrease chlorophylls biosynthetic and the effectiveness of photosynthetic in Arabidopsis and common bean (*Phaseolus vulgaris*), respectively (Priester et al. 2017; Raskar and Laware 2014). A controlled supply of zinc to plants can increase chlorophyll content, but a long-term supply can lead to toxicity and consequent decreases in plant chlorophyll content (Ahmed et al. 2009).

Plants accumulate proline and glycine betaine contents to attenuate the stressful regime by gaining growth and mediate metabolism. These processes are multifaceted and involve redox balance, osmo-protectant, and impediment of free radicals buildup, among other mechanism (Zeeshan et al. 2021). Current evidence revealed that both of gamma irradiation and ZnO-NPs enhanced the accumulation of proline compared to control (Fig. 2). It was confirmed that when soybean plants treated with ZnO-NPs displayed a higher accumulation of proline compared to soybean control plants (Zeeshan et al. 2021). Also, suggested that zinc and selenium oxide nanoparticles may be a solution to ameliorate arsenic toxicity in agricultural soil and crop plants. Proline is a signaling molecule essential for recovering plants from environment stress due to the heightened expression of the GmP5CS gene. Similarly, increased proline and glycine in chickpea (*Cicer arietinum*) upon mercury stress (Ahmad et al. 2018). Other heavy metals like cadmium in wheat (*Triticum aestivum*) (Rizwan et al. 2019), nickel in soybean (*Glycine max*) (Sirhindi et al. 2016), and chromium in chickpea (*Cicer arietinum*) elevated proline content as well (Singh et al. 2020). The combination of ZnO–NPs and/or Se-NPs improved the proline and glycine betaine contents in sesban (*Sesbania sesban*) as-stressed tissue and improved the expression of GmP5CS. Furthermore, it is well-known that proline and GB keep RUBISCO's carboxylation efficiency high under stress, eradicating oxidative damage and enhancing photosynthetic efficiency (Sivakumar et al. 2000). As well as gamma-rays promoted proline content in plants as previously established by several studies (Aly et al. 2023b; Aly et al. 2018; El-Beltagi et al. 2013).

The leaves anatomy of spinach is affected by gamma-ray (60 Gy) and ZnO-NPs used different concentration as provided in the current study (Table 4 and Fig. 3). High rate of gamma irradiation dose levels; 75, 90, as well as 105 Gy, generates bigger palisades, sponge, and upper epidermis than the control plants, respectively (Rosmala et al. 2016). A correlation among increased radiation rates and changes in leaves anatomy and phytochemical concentration of handeuleum was also shown. It was indicated that phloem and xylem of the vascular system were both expanded and differentiated and included more fibers having thickened cell walls (Tirani et al. 2019). In addition, epidermal cells were smaller and less rounded than in the control samples (Rajput et al. 2021). The parenchyma was generally denser than in the control sample, but local large areas of the intercellular space were encountered in the barely grown in 2000 mg/l ZnO–NPs (Vafaie Moghadam et al. 2022). These changes were greater at higher doses of ZnO and were greater in nano-treated samples compared bulk supplied ones. A suitable concentration of ZnO–NPs promoted the contents of vital nutrients in *Datura Stramonium* (Babajani et al. 2019) and *Melissa officinalis* NPs (Vafaie Moghadam, et al. 2022).

The SCoT markers were performed to evaluate molecular alterations in spinach plants induced by soaking seeds in ZnO–NPs at 0.0, 50, 100, and 200 ppm with or without gamma irradiation at 60 Gy. These markers are multilocus sequences useful in realizing high genetic polymorphism and generating amplicons that can be translated to gene target-specific marker systems (Xiong et al. 2011). The recent invistigation established the banding patterns and DNA profiles generated by SCoT molecular marker technique (Figs. 4, 5 as well as Table 6),

Genomic DNA damage is associated with genetic variations which lead to the modification of the binding site and changes in PCR product patterns (EL-shaer and Ibrahim 2021). Therefore, the appearance or disappearance of amplicons could be attributed to molecular alterations or mutations in binding sites. The presence of variants (polymorphism) in a sample may be assessed by the difference in its genotypes, alleles, haplotypes, or nucleotides (De Vicente et al. 2004).

The above-obtained results suggested that the SCoT marker can be used to effectively detect the molecular alterations among treated spinach plants. These results are in harmony with previously obtained in *Mangiferea indica* (Luo et al. 2010), in *Arachis hypogaea* (Xiong et al. 2011), in Echinacea (Jedrzejczyk 2020), in *Vitis uinifera* (Ahmed et al. 2021), in Lamiaceae species (Ahmed et al. 2022).

The SCoT molecular marker technique is effective in assessing genetic variations between control and mutants caused by gamma irradiation-induced alterations at the molecular, and phenotypic levels. It was found that in cowpea (*Vigna unguiculata*) the presence and loss of amplicons in the mutant genotypes compared to their untreated type proved the influence of gamma-rays on phenotypic and genotypic changeability (Vanmathi et al. 2022).

Altereds in DNA patterns as a result of ZnO–NPs applications may be induced by direct and/or indirect effects of NPs causing genotoxicity. Several studies demonstrated that ZnO–NPs indirectly cause genotoxicity through their reaction with mitochondria by promoting intracellulars levels of reactive oxygen species (ROS). Where the production of ROS in turn induces indirect DNA damage. While the direct effect appeared as NPs pass through the nuclear pore and their interact with DNA, centromere, centioles, and histone proteins through direct chemical or physical interaction (Mehrian and De Lima 2016). The production of reactive oxygen species by the plant as influenced by nanoparticles stress can lead to DNA damage and proteins oxidation with several impacts which can result in DNA alterations and thereby, it affects genetic stability (Plaksenkova et al. 2020). Besides that γ-rays is used to disinfect agriculture product for increasing shelf-life (Al-Harbi et al. 2019). Where, γ-rays induced different types of of DNA damge due to their interaction with cellular DNA directly by deposition of energy in cells and/or indirectly generating free radicals like ROS species. This DNA damage include single and double DNA strands break, base-pair deletion or insertion of base-pairs break, and DNA cross-linking that attrebuted to critical DNA damage and chromosome breaks (Nicol and Willey 2018). Add to that, the spray application of bio-derived zinc nanoparticles confidently manipulate the transcriptome and proteins profile resulting in promoting plants growth and developments (Sohail et al. 2022).

However, the results showed that the soaking in ZnO-NPs helped reduce molecular alteration rate, both spontaneous and induced by gamma irradiation. A major goal of crops improving programs is to induce differences and detect the variant at the phenotypic and genotypic rates in crops plant. So, the analysis of SCoT marker in conjunction mutagenesis will help create new spinach cultivars that perform better in terms of economic features.

Therefore, additional researches are required to demonstrate the role of Zn-ONPs as the prospective nano-protecting agent to reduce the genetic damages caused by radiation.

## Conclusion

The using of ZnO–NPs as nano-fertilizer in combination with gamma-rays for spinach plant has never been reported before. The results of the current study suggest that ZnO–NPs can be utilized as nano-fertilizers alone or in combination with gamma irradiation (60 Gy) to promote the growth of spinach plant. According to the study's findings, ZnO–NPs are effective nano-fertilizers that have a good influence on the morphological and physiological traits of spinach plant., It was found that the germination percentage enhanced to 92% and by 100 ppm ZnO–NPs while the plant length, chlorophylls and carotenoids content recorded the greatest values by the treatment 100 ppm ZnO–NPs + 60 Gy. Meanwhile, the irradiation dose level (60 Gy) combined with ZnO–NPs 200 ppm provided its maximum increase (1.069 mg/g FW) for proline content. Moreover, the anatomical studies declared that the leave epidermal tissue increased with 200 ppm ZnO–NPs in both the upper and lower epidermis. Molecular markers targeted by SCoT primers in the recent study that refer to genetic alterations caused by soaking seeds in ZnO-NPs with or without irradiation. Ten SCoT-primers succeeded in amplifying a total of 129 amplicons of which 123 were reproducible polymorphic amplicons by 94.9% with a range between 188 and 4657 bp. A total of these markers 55, with different molecular sizes, 31 of them are positive and 24 are negative. Where the lowest value of PIT % was when soaking seeds in 200 ppm (48.3%). In contrast, irradiation led to an increase in the PIT % with increasing ZnO-NPs concentrations upto 100 ppm (65.3%). whilst, the effect of soaking at 200 ppm was beneficial in decreasing the value of PIT % (to 51.9%), It could be recommended to apply ZnO–NPs for plant improvement without compromising the morphological as well as nutritional qualities. The appropriate application of ZnO–NPs would also help to improve the nutritional value of crop products.
